# Internal Hernia Through Acquired Broad Ligament Defect: A Case Report

**DOI:** 10.7759/cureus.66766

**Published:** 2024-08-13

**Authors:** Anagha T R, Noorul Hassan, Manikanta K S

**Affiliations:** 1 General Surgery, Bangalore Medical College and Research Institute, Bangalore, IND

**Keywords:** small bowel obstruction, abdominopelvic computed tomography, exploratory laparotomy, internal hernia, broad ligament

## Abstract

Internal hernias are relatively uncommon occurrences in cases of mechanical bowel obstructions. They occur when the small bowel herniates through a recess or defect within the abdominal cavity. Herniation through a defect in the broad ligament is particularly rare among internal herniations.

We present the case of an 88-year-old female who presented to the emergency department with a history of abdominal pain and obstipation. The patient had undergone open tubectomy 43 years ago. Erect abdominal radiograph and contrast-enhanced computed tomography confirmed the presence of intestinal obstruction. Exploratory laparotomy revealed a viable small intestinal loop herniating through a defect in the right broad ligament. The herniated bowel loop was reduced, and the defect was closed. The contralateral side was examined to confirm the absence of defects in the left broad ligament.

Early diagnosis of internal hernia through broad ligament defect requires a high index of suspicion, and the advent of computed tomography has facilitated early preoperative diagnosis. Rapid management is necessary to prevent catastrophic sequelae such as strangulation and gangrenous changes in the herniated bowel.

## Introduction

Intestinal obstruction of various types is a common clinical entity encountered in emergency setting. Internal hernias are relatively rare and are reported in 1-2% of mechanical bowel obstructions. They occur when abdominal viscera herniate through a recess or defect within the peritoneum or mesentery.

The broad ligament is a peritoneal fold attaching the fallopian tubes, ovaries, and uterus to the wall and floor of the pelvis. Herniation through a defect in the broad ligament is quite rare and accounts for 4% of internal herniations [1]. The internal hernias are challenging to diagnose both clinically and radiologically due to the vague symptoms of presentation and nonspecific findings on the erect abdominal radiograph. The use of computed tomography has enabled early preoperative diagnosis and management possible, thereby reducing associated morbidity and mortality [2].

We present a case of an 88-year-old female with small bowel obstruction diagnosed with internal hernia through a right broad ligament defect. This case report follows the Surgical CAse REport (SCARE) Guidelines [3].

## Case presentation

An 88-year-old multiparous female presented to the emergency department with abdominal pain for five days associated with obstipation and bilious vomiting since three days. Pain was colicky in nature and diffuse, aggravated on consumption of food. She has no known comorbidities. The patient had a history of four normal vaginal deliveries and had undergone open tubectomy 43 years ago.

On examination, she was found to be normotensive with tachycardia and dry buccal mucosa. Per abdomen examination revealed a mildly distended abdomen with diffuse tenderness and guarding. Laboratory reports revealed total white blood cell count of 17,400 cells per cubic millimeter with neutrophil percentage of 90% and a hemoglobin level of 11.1 gram per deciliter. Renal function, serum electrolytes, and liver function tests were within normal limits. Erect abdominal radiograph showed dilated large bowel loops. Contrast-enhanced computed tomography of the abdomen and pelvis showed dilated distal small bowel loops with transition point located on the right side of the uterus, and a mild collection was also noted in the peritoneal cavity.

**Figure 1:**
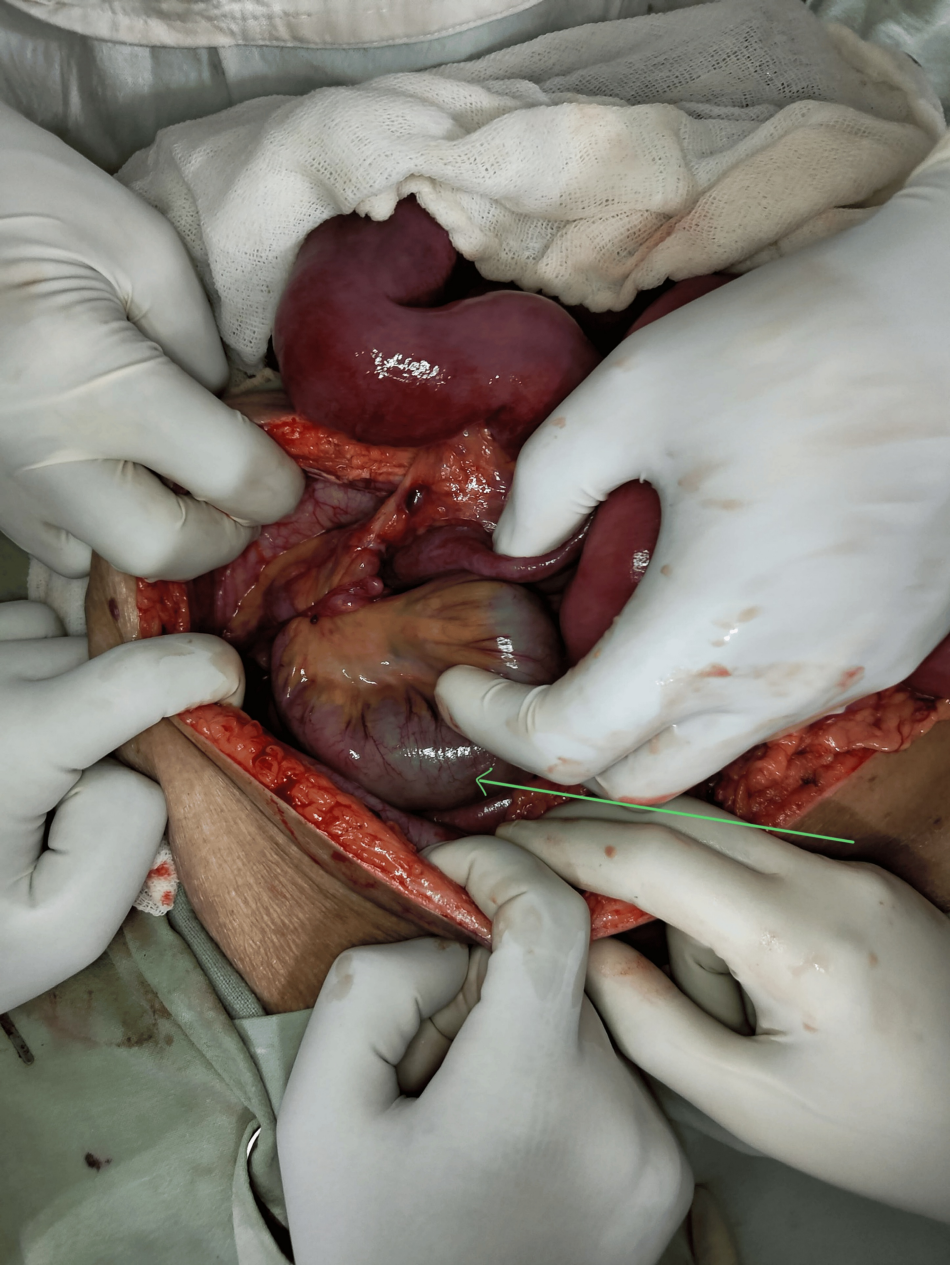
Ischemic viable small bowel loop herniating through the broad ligament defect as shown by the green arrow.

The patient underwent exploratory laparotomy, which revealed a defect of 5x4 centimeters in the right broad ligament with a herniating loop of small bowel located 15 centimeters from the ileocecal junction. The bowel loops were found to be ischemic but viable after adhesiolysis and reduction of the hernial contents (Figure 1). The broad ligament defect was closed with polydioxanone 2-0 continuous sutures. The left broad ligament was inspected and was found to be intact. The patient tolerated the procedure well and was started on oral intake on postoperative day 3.

## Discussion

Internal hernias are classified according to the location of the hernia orifice. These include paraduodenal, intersigmoid, pericecal, transmesenteric, foramen of Winslow, and transomental hernias. Paraduodenal hernias are more frequent, while broad ligament hernias are relatively rare.

**Figure 2:**
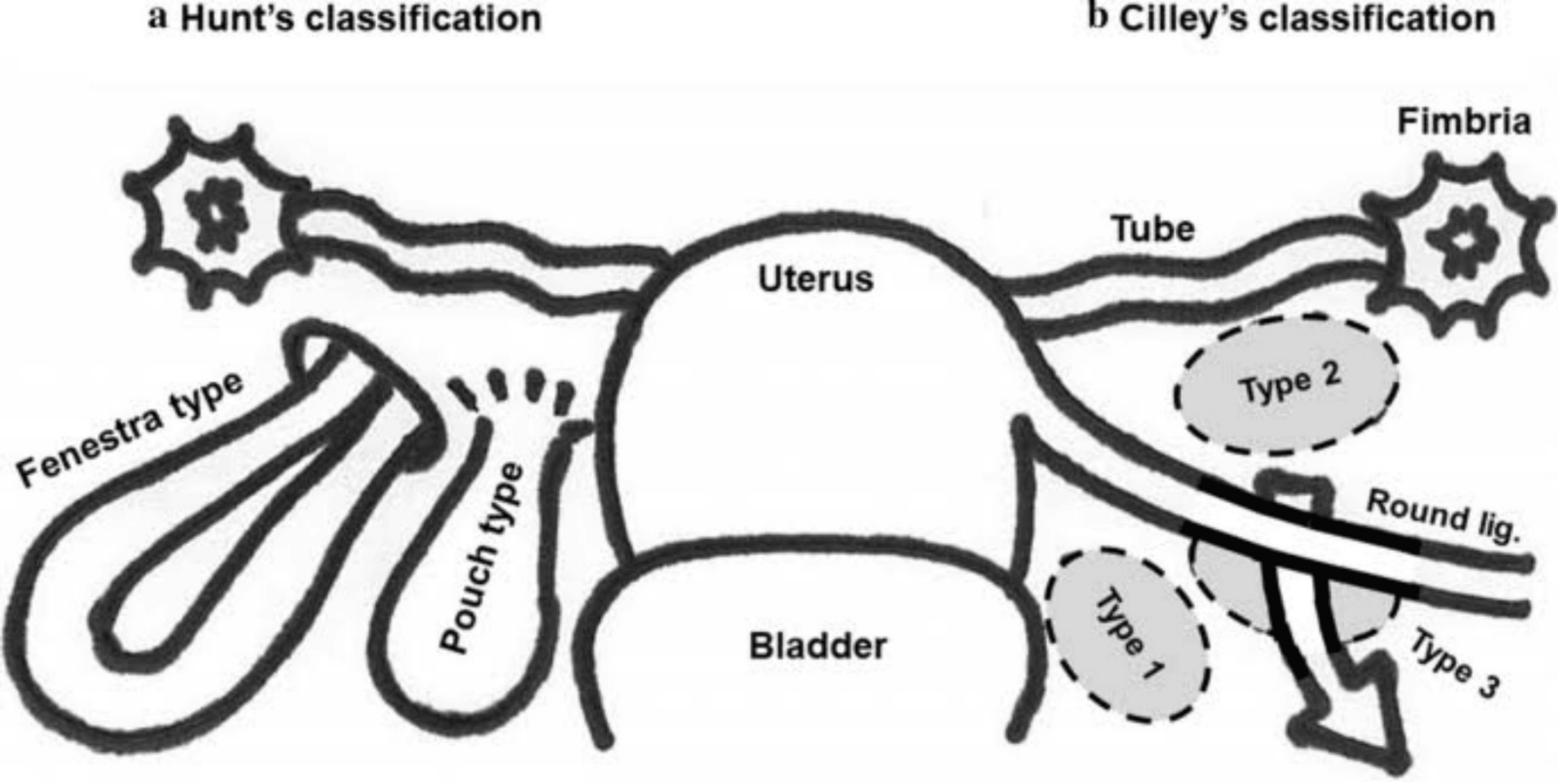
Hunt’s and Cilley’s classification. Source: [6]

The first reported case of a broad ligament hernia was in 1861 by Quain. The autopsy of the woman showed a right broad ligament defect with strangulation of the bowel [4]. The broad ligament defects were classified by Hunt according to the degree of peritoneal defect: a) pouch type, where one layer of broad ligament has defect, and b) fenestra type, where both peritoneal layers have defects. Cilley classified broad ligament hernia according to the location of the defect: a) type 1 defects occur caudal to the round ligament, b) type 2 defects occur above the round ligament of the uterus, and c) type 3 defects occur in the mesoligamentum teres of the uterus [5]. Our case was fenestra type 2 ( Figure 2).

The etiology of broad ligament defects can be congenital or acquired. Acquired factors include operative trauma, pregnancy, perforations during vaginal manipulation, prior pelvic inflammatory disease, or endometriosis. Congenital cases involve developmental defects around the uterus, such as spontaneous rupture of cystic structures [7].

Imaging studies play an important role in the diagnosis of broad ligament hernias owing to vague clinical presentation. Computed tomography has become the first-line imaging technique. Barium studies show distended small bowel loops in the pelvis, crowding of small bowel loops in the hernial sac, and obstruction suggested by segmental dilatation and stasis. Additionally, mesentric vessel engorgement, crowding, twisting, and stretching also provide important clues to diagnosis.

Broad ligament hernias, though a rare occurrence, can be rapidly fatal if not diagnosed and managed appropriately. Surgery remains the mainstay of management. The principles of surgical intervention include early intervention to prevent bowel strangulation, reduction of the hernia, resection of any non-viable part of the bowel, and closure of the defect. It is imperative to visualize the contralateral broad ligament to identify any defects that could lead to recurrence of intestinal obstruction [8]. There have been considerable advances in surgical management, including single-incision laparoscopic surgery and needlescopic surgery. The mode of surgery performed is determined based on the patient’s condition and the surgical expertise available.

## Conclusions

This case report emphasizes the need to consider broad ligament hernia as an important differential diagnosis in women presenting with acute intestinal obstruction, especially in multiparous women with prior surgical history. Early diagnosis has been possible with advent of multidetector computed tomography. Timely decisions regarding surgical management, either by laparoscopy or laparotomy, are necessary to prevent catastrophic sequelae.
